# A role for orphan nuclear receptor liver receptor homolog-1 (LRH-1, NR5A2) in primordial follicle activation

**DOI:** 10.1038/s41598-020-80178-4

**Published:** 2021-01-13

**Authors:** Marie-Charlotte Meinsohn, Camilla H. K. Hughes, Anthony Estienne, Hatice D. Saatcioglu, David Pépin, Raj Duggavathi, Bruce D. Murphy

**Affiliations:** 1 Centre de recherche en reproduction et fertilité, Université de Montréal, 3200 rue Sicotte, St-Hyacinthe, QC J2S 7C6 Canada; 2 Pediatric Surgical Research Laboratories, Simches Research Center, Massachusetts General Hospital, 185 Cambridge St., Boston, MA 02114 USA; 3 Department of Animal Science, McGill University, 21111 Lakeshore Rd., MS1085, Ste-Anne de Bellevue, QC H9X 3V9 Canada

**Keywords:** Developmental biology, Physiology, Endocrinology

## Abstract

Liver receptor homolog-1 (NR5A2) is expressed specifically in granulosa cells of developing ovarian follicles where it regulates the late stages of follicle development and ovulation. To establish its effects earlier in the trajectory of follicular development, NR5A2 was depleted from granulosa cells of murine primordial and primary follicles. Follicle populations were enumerated in neonates at postnatal day 4 (PND4) coinciding with the end of the formation of the primordial follicle pool. The frequency of primordial follicles in PND4 conditional knockout (cKO) ovaries was greater and primary follicles were substantially fewer relative to control (CON) counterparts. Ten-day in vitro culture of PND4 ovaries recapitulated in vivo findings and indicated that CON mice developed primary follicles in the ovarian medulla to a greater extent than did cKO animals. Two subsets of primordial follicles were observed in wildtype ovaries: one that expressed NR5A2 and the second in which the transcript was absent. Neither expressed the mitotic marker. KI-67, indicating their developmental quiescence. RNA sequencing on PND4 demonstrated that loss of NR5A2 induced changes in 432 transcripts, including quiescence markers, inhibitors of follicle activation, and regulators of cellular migration and epithelial-to-mesenchymal transition. These experiments suggest that NR5A2 expression poises primordial follicles for entry into the developing pool.

## Introduction

The development of the mouse ovary begins with sexual differentiation on embryonic day 10.5, characterized by mitotic replication of oogonia^1^. Most of the incipient oocytes initiate meiosis between embryonic days 13.5 and 15.5^2^. Many are lost, but those that persist can be found densely packed in structures known as germ cell cysts^3^. Beginning on approximately day 19.5 of gestation, these cysts undergo a programmed breakdown, when the pre-granulosa cells invade and surround each oocyte, forming the primordial follicles^4^. This process continues through the first four to six days of postnatal life, and oocytes that fail to be encapsulated succumb to apoptosis^5^. In the mouse, approximately 33% of the oocytes survive to form the primordial follicles^4^. The consensus view is that this process of primordial follicle formation establishes the ovarian reserve that will provide oocytes throughout reproductive life^6^.

Although enormous progress has been made in understanding the events and regulation of the trajectory of ovarian follicular development, the early stages of development remain, to a large extent, a mystery^7^. The majority of the follicles in the reserve are dormant. It has been shown that the maintenance of primordial follicles in this quiescent state is not a passive process, but that the resting pool of primordial follicles is under a constant inhibitory influence^8,9^. Nonetheless, once activated, continuation of development is an irreversible process, and follicles that have initiated growth undergo atresia if not selected for subsequent stages of maturation^10^. Activation of primordial follicles occurs via a gonadotropin-independent process, whereby they are gradually selected from the quiescent reserve into the growing follicle pool^11,12^. Extensive bidirectional signaling takes place between oocytes and granulosa cells to ensure follicle development from primordial stage onwards^13,14^. As oocytes begin to increase in size, the granulosa cells undergo a partial epithelial-to-mesenchymal transition under the control of ZEB, SNAIL and TWIST family members^15^. This transition engenders change in the shape of these somatic cells from flattened to cuboidal, and presages the increase in their proliferation and formation of multiple layers^16^. Several activator and repressor signaling pathways have been implicated in the control of primordial follicle activation, as indicated by the patterns of expression of the oocyte-specific factors GDF9, FOXO3 and PTEN as well as the granulosa cell-specific FOXL2^17^.

Recent studies have shown that the orphan nuclear receptor liver receptor homolog 1 (LRH-1, NR5A2) influences follicular development and has an impact on multiple key follicular processes, including granulosa cell proliferation^18^. NR5A2 expression is restricted to the granulosa cells of primordial and all subsequent follicles in the ovary, and to luteal cells^19^. Germline deletion of NR5A2 results in early embryo lethality^20^, thus, a conditional attenuation strategy is necessary for exploration of its role in the ovary. We have previously shown by means of granulosa cell-specific depletion, beginning at either the primordial or antral follicle stage or in the incipient corpus luteum, that NR5A2 is required, not only for successful follicle development and subsequent ovulation, but also for pregnancy^21–24^. To date, there is not a great deal of information about the developmental dynamics of primordial follicles under physiological conditions. It is known that NR5A2 is expressed in this follicle population in the human ovary^25^ but no information has emerged on its role in either primordial follicle activation or maintenance of the quiescence of the follicle reserve.

The objectives of the current study were to explore the role of NR5A2 in ovarian function, with focus on its contribution to primordial follicle activation. We used a mutant mouse model in which NR5A2 is depleted from the granulosa cells of follicles at all stages, from primordial follicles forward. Our results demonstrate a role for NR5A2 in the regulation of primordial follicle activation.

## Results

### NR5A2 mRNA and proteins are depleted in conditional (cKO) mice at the termination of primordial follicle formation

**Figure 1:**
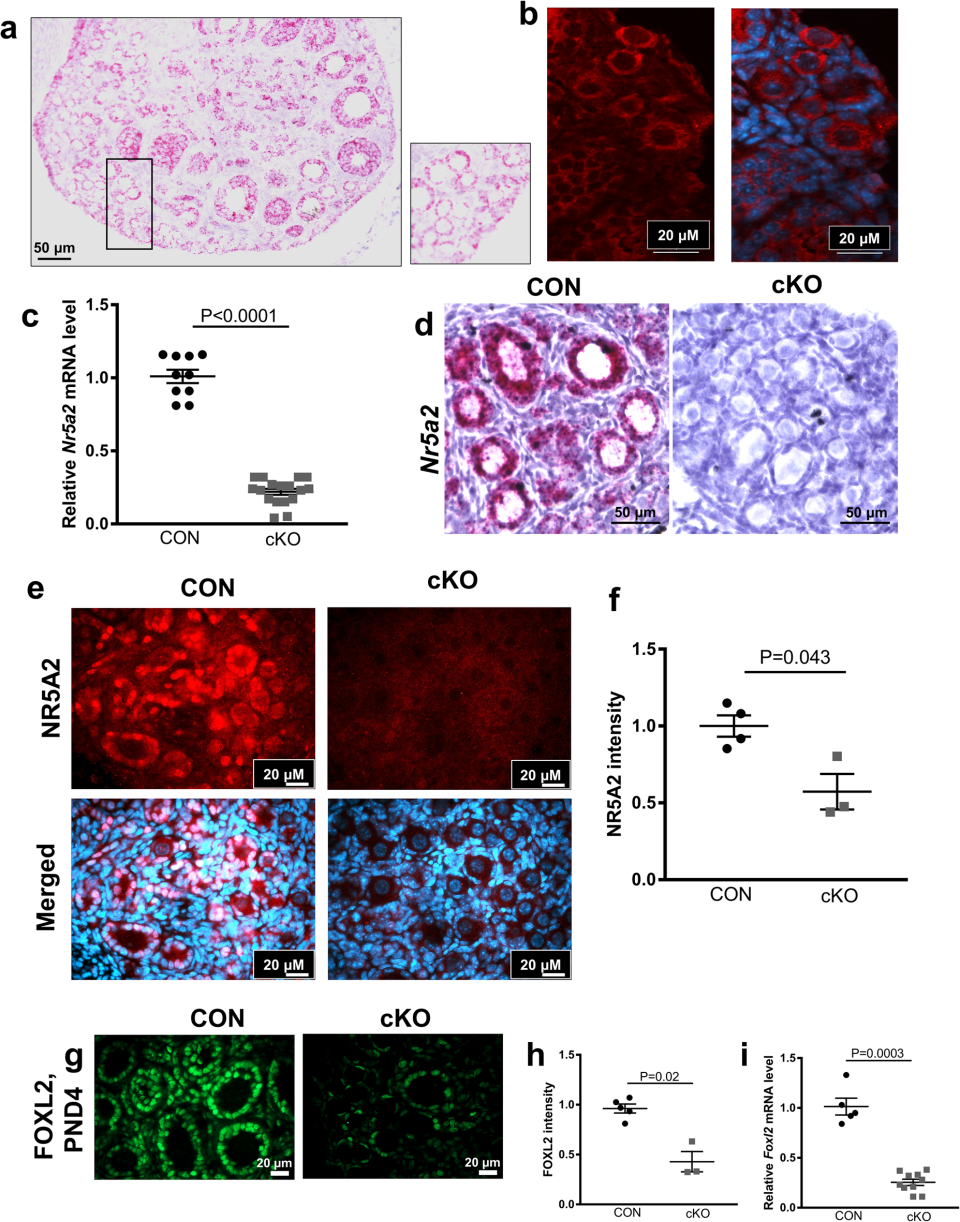
Depletion of NR5A2 from knock-out (cKO) ovaries at the termination of primordial follicle formation, via AMRH2-cre. (**a**) RNA in situ hybridization of *Amhr2* in CON ovaries at PND6. (**b**) Immunolocalization of AMHR2 in the ovaries of PND13 CON mice. (**c**) Abundance of *Nr5a2* transcripts in CON and cKO mouse ovaries at PND4 (n = 5–10 per genotype). (**d**) RNA in situ hybridization of *Nr5a2* in CON and cKO ovaries at PND6. (**e**) Immunolocalization of NR5A2 in PND4 CON and cKO mouse ovaries. Quantitative analysis of NR5A2 expression in CON and cKO PND4 mouse ovaries (n = 3–6 animals per genotype). (**f**) Quantification of signal from immunofluorescence in CON and cKO mouse ovaries on PND4. (**g**) Localization of FOXL2 in CON and cKO mice ovaries at PND4 showing absence of FOXL2 signal in primordial follicles of the cKO mouse. (**h**) Quantitative analysis of FOXL2 expression between CON and cKO PND4 mice ovaries (n = 3–5 animals per genotype). (**i**) Abundance of *Foxl2* transcripts in CON and cKO mice ovaries at PND4 (n = 5–10 animals per genotype). Quantification or protein expression for NR5A2 and FOXL2 was performed using CellProfiler version 4.06, www.cellprofiler.org.

To confirm the depletion of NR5A2 from the neonatal (PND4) and PND13 ovaries, we first demonstrated by in situ hybridization and immunohistochemistry that AMHR2 is expressed in pre-granulosa cells of the primordial follicle. The *Amhr2* transcript localized to the granulosa cells of primordial, primary and secondary follicles in the immature ovary, as well as to the surface epithelium (Fig. 1a). Immunohistochemistry further confirmed the AMHR2 protein to be present in these three classes of follicles (Fig. 1b). Together these findings provide evidence for Cre/lox recombination at least as early as the primordial follicle stage of the follicle development trajectory. We then substantiated the depletion of NR5A2 from perinatal ovaries between PND3 and PND6, the period of termination of primordial follicle formation, by qPCR, in situ hybridization and immunohistochemistry (Fig. 1c–f). Whole ovary qPCR revealed 80% decrease (*p* < 0.0001) in *Nr5a2* mRNA abundance in the cKO mice (Fig. 1c). In keeping with the reduction seen by qPCR, *Nr5a2* mRNA in cKO ovaries at PND6 was undetectable by in situ hybridization (RNAScope; Fig. 1d). Immunohistochemical evaluation of NR5A2 protein demonstrated the expected nuclear localization of NR5A2 in CON ovaries (Fig. 1e). Little signal was observable in primordial and primary follicles in the cKO ovaries, as confirmed by a significant reduction in fluorescence, detectable by CellProfiler software (Fig. 1f).

Forkhead box protein L2 (FOXL2) is a granulosa cell-specific winged-helix transcription factor that is required for activation of the primordial follicle to the primary state^26^. In a recent publication^27^ it was shown that this factor is expressed differentially between subpopulations of pregranulosa cells during primordial follicle formation. We hypothesized that if NR5A2 could elicit its effect on follicular populations by regulating abundance of this key transcription factor, it could also serve as a marker for the efficacy of depletion of the nuclear receptor. Comparison of FOXL2 mRNA and protein abundance in cKO and CON ovaries at PND4 revealed that its expression was markedly lower in the NR5A2 depleted ovaries (Fig. 1 g–i).

### Phenotypic differences between the cKO and CON ovaries at postnatal day 4

**Figure 2:**
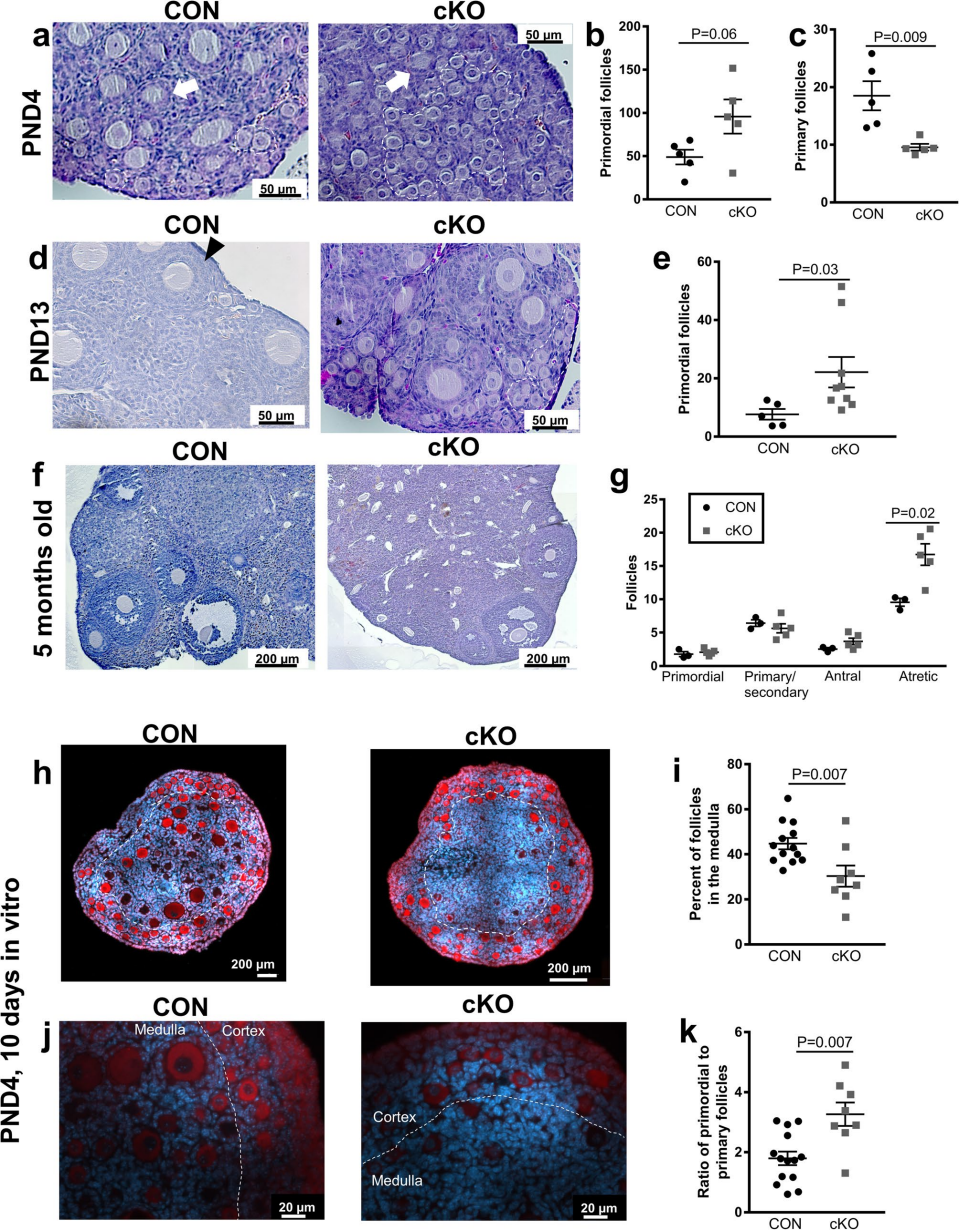
Increased number of primordial follicles in immature NR5A2 knock-out (cKO) mice. (**a**) Hematoxylin/Eosin staining of postnatal day 4 (PND4) control and cKO mouse ovaries showed an increased number of primordial follicles and a decreased number of primary follicles in cKO compared to control. The populations of primordial (**b**) and primary (**c**) follicles on PND4 were quantified (n = 5 animals per genotype). (**d**) Hematoxylin/Eosin staining analysis showed that the number of primordial follicles was also increased in cKO mouse ovaries at PND13 compared to the CON. The primordial follicles are located principally in the cortices of the ovaries. (**e**) The population of primordial follicles on PND13 was enumerated (n = 5–9 animals per genotype). (**f**) Histological analysis of mature (5-month-old) ovaries in cKO and CON animals. (**g**) The populations of primordial, primary/secondary, antral and atretic follicles were quantified (n = 3–5 animals per genotype). In a, b, d, and e, example primordial follicles groups are circled with a dashed line. In d, one secondary follicle is marked with a black triangle. In (**a**,**b**), example primary follicles are marked by a white arrow. (**h**,**j**) CON and cKO mouse ovaries cultured for 10 days in vitro, at low (**h**) and high (**j**) magnification. Oocytes are stained with antibodies to DDX4 (red). (**i**) Quantification of the follicles in the medulla of cultured ovaries (n = 8–13 animals per group). (**k**) Quantification of the primordial follicle population in cultured ovaries.

Histological evaluation of ovaries of mice at PND4, when the follicle population is limited to primordial and primary follicles^28^ confirmed the presence of these two follicle subtypes in both CON and cKO mice (Fig. 2a). At PND4, we observed a larger number of primordial follicles, but fewer primary follicles in the cKO ovary compared to the control mice (Fig. 2a–c). This increase was also present at the time of appearance of the first antral follicles, on PND13 (Fig. 2d,e)^28^.

The number of primordial follicles in five-month-old cKO mice relative to CON was similar. In contrast, the number of atretic follicles in the cKO ovary, identified by the presence of a degenerate oocyte, was consistently and substantially greater than found in CON mice (Fig. 2f,g).

To further study the role of NR5A2 in follicular development, we established an organ culture model of postnatal day 4 (PND4) ovaries, incubated over 10 days. We identified oocytes by localization of the gamete specific factor, DDX4 (red; Fig. 2h,j). In CON mice we observed primordial follicles in the cortex and numerous developing primary follicles, mainly located in the medulla, consistent with previously published studies^29^. Moreover, the number of developing follicles in the medullary zone of the ovary in cKO mice was significantly lower (Fig. 2i). Further, in the cKO ovaries, the ratio of primordial to primary follicles was substantially greater relative to the CON (Fig. 2k), in keeping with in vivo results.

### NR5A2 defines two subsets of primordial follicles

**Figure 3:**
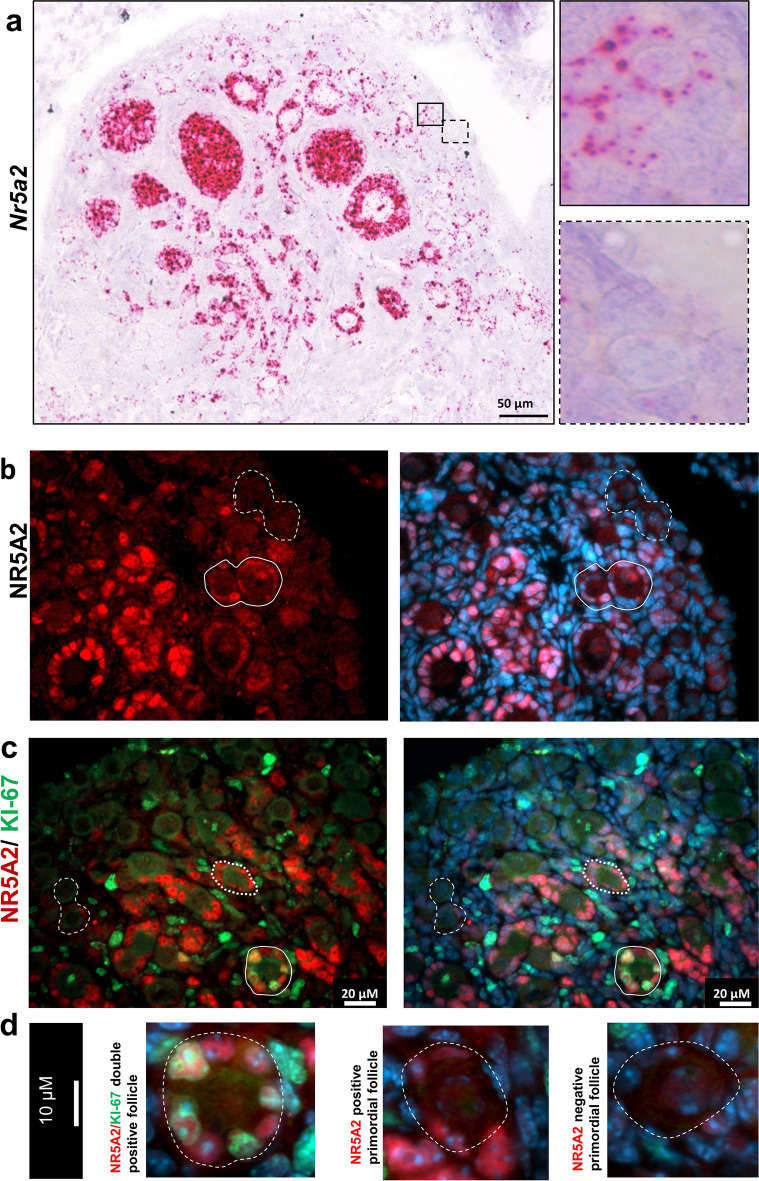
NR5A2 defines two subsets of primordial follicles. (**a**) RNA in situ hybridization of *Nr5a2* in CON mouse ovaries at PND6. Panels at right demonstrate two types of primordial follicles, one that expresses NR5A2 (red signal). (**b**) Immunolocalization of NR5A2 in the ovaries of PND3 CON mice. The left panel displays immunolocalization of NR5A2 (red), while the right panel shows NR5A2 and the nuclear stain, DAPI (blue). The dashed line indicates follicles lacking NR5A2, while the solid line designates follicles with NR5A2 expression. To the right are larger images of the example follicles of interest. (**c**) Immunolocalization of NR5A2 (red) and KI-67 (green) in the ovaries of PND3 CON mice. The dashed line indicates primordial follicles without the NR5A2 signal, the dotted line indicates a primordial follicle expressing NR5A2 but not KI-67, and the solid line indicates primary follicles expressing both NR5A2 and KI67. (**d**) High magnification images of (from left to right) an NR5A2, KI67 double positive primary follicle, an NR5A2 positive, KI67 negative primordial follicle, and an NR5A2, KI67 negative follicle.

To investigate the role of NR5A2 in primordial to primary follicle transition, we performed RNA in situ hybridization and fluorescent immunocytochemistry in ovaries of wild type mice on PND3 and PND6. Localization of the *Nr5a2* transcript revealed the existence of two populations of primordial follicles in the neonatal mouse ovary, one expressing NR5A2 and a second that was devoid of the signal (Fig. 3a,b,d). To further corroborate the observation that the follicles expressing NR5A2 have not yet been activated, we performed a double immunofluorescence to detect NR5A2 and KI-67, a cellular proliferation marker (Fig. 3c,d). As expected, we found that activated primary follicles expressed both NR5A2 and KI-67. More significantly, there was a subpopulation of primordial follicles that expressed neither NR5A2 nor KI-67, as well as a demonstrable subset of primordial follicles that expressed only NR5A2 but not KI-67. (Fig. 3c,d).

### Loss of NR5A2 alters abundance of transcripts associated with migration and epithelial-to-mesenchymal transition

**Figure 4:**
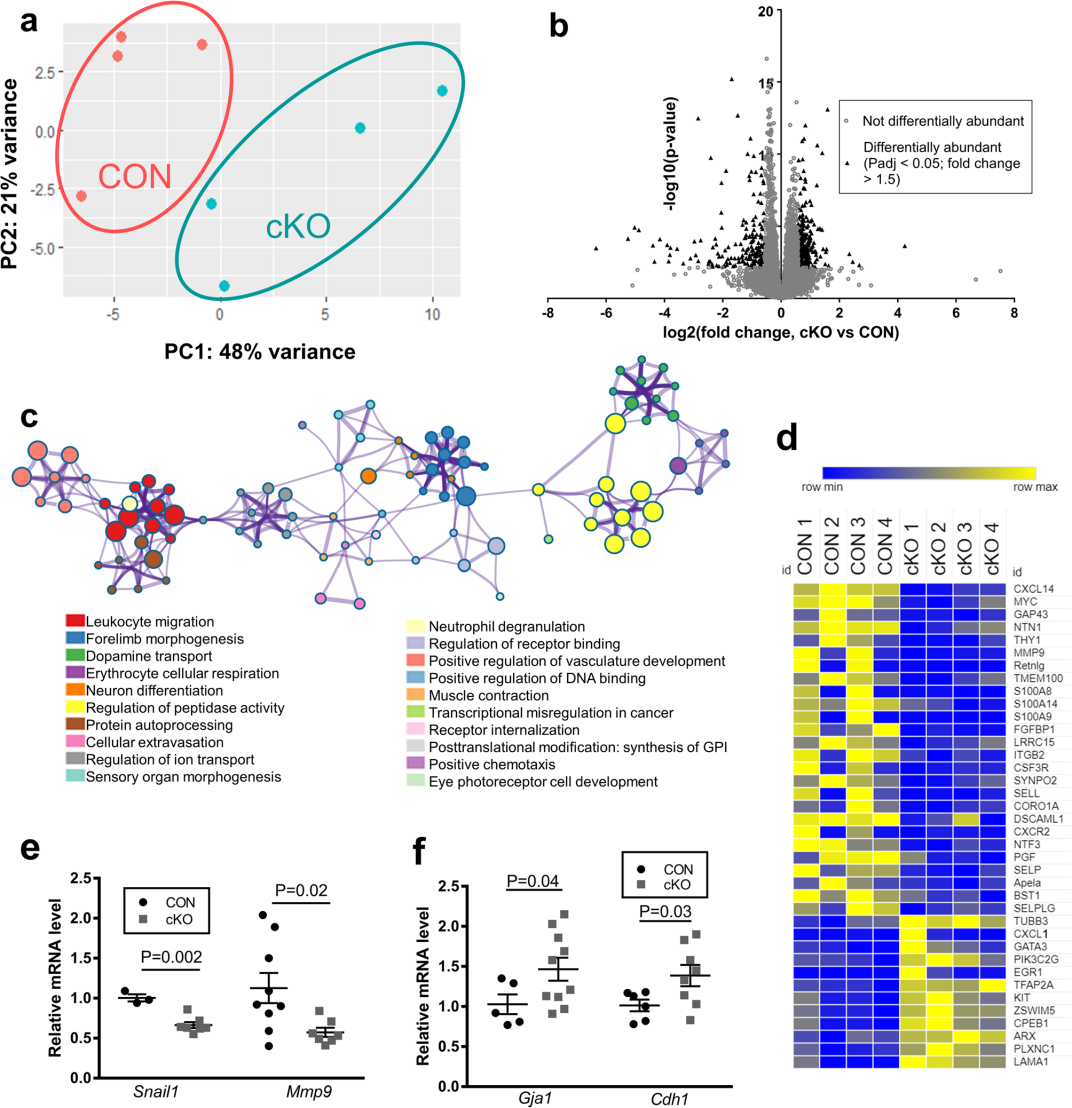
NR5A2 conditional knockout alters the ovarian transcriptome on PND4. (**a**) Principal component analysis of all transcript abundance data from CON (each sample represented by a red circle) and cKO (each sample represented by circle in teal) ovaries. (**b**) Volcano plot of all mRNA quantified and analyzed in CON and cKO ovaries. Black triangles represent transcripts with Padj < 0.05 and fold change > 1.5 (either up or down), while grey circles represent transcripts with Padj > 0.05 or fold change < 1.5. (n = 4 animals per genotype). (**c**) Pathway analysis transcripts that were downregulated by NR5A2 cKO. The color of each circle represents the functional cluster, while the edges represent the similarity or relatedness among clusters. (**d**) Heatmap of all differentially abundant transcripts related to migration (Metascape). Blue represents a decrease, while yellow represents an increase. (**e**) Abundance of markers of the epithelial to mesenchymal transition that changed in RNAseq (Padj < 0.05), as measured by qPCR, *Snail*, *Mmp9* (n = 3–10 animals per genotype). (**f**) Abundance of epithelial markers that are downstream of, and inhibited by SNAIL, *Gja1* and *Cdh1*, as measured by qPCR (n = 5–10 animals per genotype).

To generate a more comprehensive understanding of the effect of depletion of NR5A2 on follicular function on PND4, we compared transcriptomes between CON and cKO ovaries. Principal component analysis demonstrated that samples from PND4 CON and cKO ovaries clustered separately (Fig. 4a). Among 15,657 transcripts detected and analyzed, 432 were differentially abundant, with 178 downregulated and 254 upregulated in cKO ovaries (Fig. 4b; Supplementary Table S1). Validation of candidate genes related to the mechanisms that differed between the cKO and CON in RNAseq analysis was achieved by qPCR (Table 1). Eighteen transcripts were identical in pattern of change and statistical significance in RNAseq and qPCR, and an additional eight transcripts tended to change (*p* < 0.05, but padj > 0.05) in RNAseq, but were confirmed as significantly changed in qPCR. Changes in transcript abundance of only five genes from RNAseq (padj < 0.05) proved undetectable by qPCR (*p* < 0.05), and all of these had consistent fold changes in RNAseq and qPCR (Table 1).

**Table 1:** Abundance of transcripts in CON and cKO ovaries as measured by RNAseq and qPCR.

mRNA	Fold change (RNAseq)	Fold change (qPCR)	*p* value (RNAseq)	*p* value (qPCR)	padj (RNAseq)
*Rgs11*	1.24	1.17	2.4E−11	0.001	1.0E−08
*Grem2*	0.44	0.38	1.9E−07	0.08	1.9E−05
*Kcnj3*	1.04	1.37	3.2E−07	0.0001	3.0E−05
*cKit*	0.63	0.87	6.4E−05	0.05	0.002
*Smad3*	0.42	0.54	7.0E−05	0.006	0.002
*Mmp9*	− 2.26	− 0.98	7.1E−05	0.02	0.002
*Gdf9*	0.72	0.45	8.5E−05	0.10	0.002
*Amhr2*	− 0.59	− 0.77	0.0002	0.05	0.004
*Fos*	4.24	0.79	0.0002	0.13	0.005
*p63*	0.59	0.33	0.0005	0.45	0.008
*Egr1*	2.34	0.65	0.0008	0.03	0.01
*Foxo3*	0.33	1.09	0.002	0.0001	0.02
*Snail1*	− 0.44	− 0.59	0.002	0.002	0.02
*Cxcl1*	2.68	0.93	0.005	0.05	0.04
*Figla*	0.53	0.85	0.007	0.03	0.05
*Notch2*	0.30	0.54	0.008	0.01	0.05
*Twist1*	− 0.33	− 0.18	0.008	0.25	0.05
*Fosb*	2.76	1.11	0.008	0.04	0.06
*Cdh2*	0.21	0.37	0.02	0.02	0.09
*Lin28a*	0.65	1.58	0.02	0.01	0.09
*Wnt4*	0.19	0.08	0.02	0.62	0.09
*Junb*	1.69	1.45	0.04	0.04	0.16
*Cxcl10*	2.30	0.58	0.04	0.04	0.17
*Smad2*	0.10	0.39	0.05	0.01	0.17
*Zfp36*	0.87	0.31	0.05	0.13	0.18
*Foxl2*	0.16	− 2.00	0.07	0.0003	0.22
*Bmpr1b*	0.15	0.94	0.09	0.0004	0.27
*Gja1*	0.14	0.51	0.09	0.04	0.27
*Kitl*	0.14	0.38	0.14	0.05	0.35
*Zeb1*	0.12	0.20	0.14	0.47	0.35
*Amh*	− 0.51	− 1.89	0.25	0.004	0.48
*Pten*	− 0.04	0.54	0.50	0.12	0.71
*Cdkn1a*	0.06	1.02	0.51	0.002	0.72
*Bcl2l1*	− 0.06	0.73	0.52	0.11	0.73
*Hmga2*	0.10	0.85	0.54	0.0008	0.74
*Zeb2*	0.05	0.59	0.61	0.008	0.79
*Cdh1*	− 0.08	0.45	0.78	0.03	0.89
*Vim*	− 0.02	0.15	0.82	0.42	0.92
*Mcl1*	0.00	0.49	1.00	0.008	1.00
*Grem1*	0.39	0.73	0.60	0.08	NA

Pathway analysis revealed that transcripts downregulated by Nr5a2 cKO were primarily associated with cell migration, including pathways for leukocyte migration, cellular extravasation, and chemotaxis (Fig. 4c). The mRNA associated with these pathways included components of cytokine and chemokine signaling (*Cxcr2*, *Cxcl14*, *A2m*, *Csf3r*), adhesion molecules (*Sell*, *Selp*, *Selplg*), and metalloproteases (*Mmp8*, *Mmp9*), among others (Fig. 4d). Pathways associated with vascular development were also disrupted, including transcripts for *Angptl7*, *Fgfbp1* and *Mmp9*. Other top pathways included those for normal cellular functions, such as ion transport, DNA binding and receptor binding, indicating that downregulated mRNA may be involved in maintaining cellular homeostasis (Fig. 4c, Supplementary Table S2). We hypothesized that, because cellular migration-related functions were negatively affected by loss of Nr5a2, this transcription factor may mediate the epithelial-to-mesenchymal transition that characterizes follicular activation^15^. Confirmation by qPCR of the decreases of mesenchymal markers *Mmp9* and *Snail1* by RNAseq supports this postulate (Fig. 4e). To provide additional evidence for modulation of the mesenchymal transcription factor *Snail1*, two of its downstream targets, *Cdh1* and *Gja1*, were also quantified by qPCR*.* Both *Cdh1* and *Gja1* have been reported to decrease during the escape from the epithelial state^30–32^. As expected, both these epithelial markers were significantly upregulated in our cKO mouse model suggesting active maintenance of the epithelial condition (Fig. 4f).

Some of the mRNA upregulated in cKO ovaries were oocyte-specific transcripts. Among the primary pathways identified as increasing in cKO were those related to meiosis (Supplementary Fig. S1, Supplementary Table S3). Interestingly, among oocyte transcripts that were in greater abundance in cKO ovaries were *Gdf9*, *Kit*, *Figla*, and *Bmp15*. The former three were evaluated by qPCR and both *Kit* and *Figla* were more abundant in cKO ovaries than in control (Supplementary Fig. S1).

### NR5A2 depletion affects quiescence gene expression in primordial follicles

**Figure 5:**
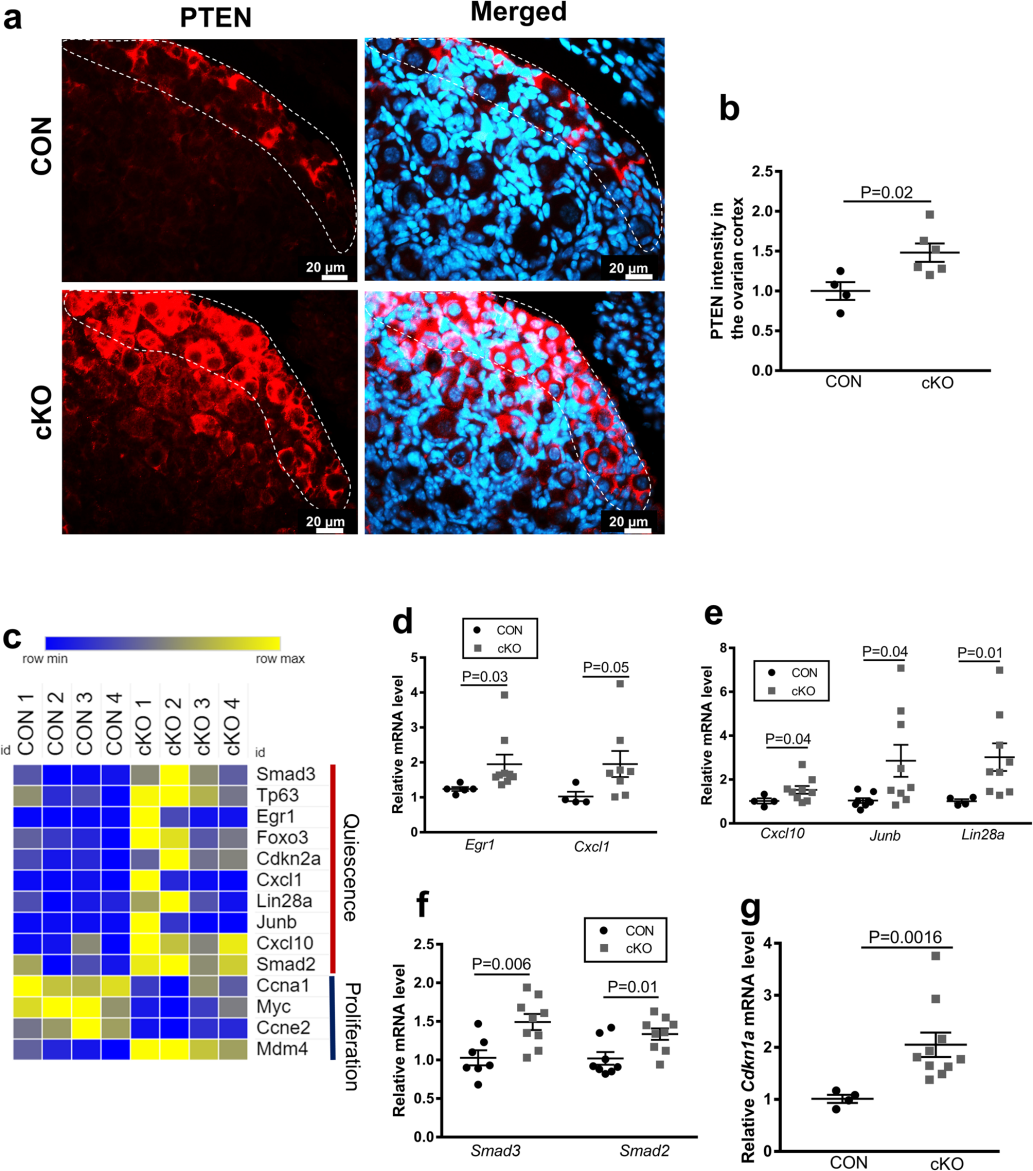
NR5A2 depletion affects follicle activation-related and quiescence pathways. (**a**) Immunolocalization of PTEN in CON and cKO mice ovaries at PND4. DAPI staining in right panel. (**b**) Quantitative analysis of PTEN expression between CON and cKO PND4 mice ovarian cortices (n = 4–6 animals per genotype). Quantification or protein expression was performed using CellProfiler version 4.06, www.cellprofiler.org. (**c**) Heatmap of all differentially abundant transcripts related to quiescence and proliferation (manually selected). Blue represents a decrease, while yellow represents an increase. (**d**) Abundance of quiescence markers that changed by RNAseq (Padj < 0.05), as measured by qPCR. Markers include *Cxcl1* and *Egr1* transcripts in CON and cKO mice ovaries at PND4 (n = 5–10 animals per genotype). (**e**) Abundance of quiescence markers that tended to change by RNAseq (*p* < 0.05), as measured by qPCR. Markers include *Cxcl10*, *Junb*, and *Lin28a* transcripts in CON and cKO mice ovaries at PND4 (n = 5–10 animals per genotype). (**f**) Abundance of *Smad3* and *Smad2*, regulators of primordial follicle quiescence, as measured by qPCR (n = 5–10 animals per genotype). *Smad3* changed by RNAseq (Padj < 0.05), while *Smad2* tended to change (*p* < 0.05). (**g**) Abundance of downstream marker of activation of quiescence pathways *Cdkn1a*, as measured by qPCR.

In the cKO relative to the CON ovary, we observed a greater expression of PTEN, which is known to be a principal factor constraining primordial follicle activation^33^ (Fig. 5a,b). As PTEN is a tumor suppressor, we hypothesized that Nr5a2 depletion would further be manifest in the expression of genes associated with proliferative quiescence. This proved true, as transcriptome analysis revealed modification in a number of these genes (Fig. 5c). Subsequent validation by qPCR confirmed the mRNA abundance of these genes was elevated in the PND4 ovaries from cKO animals (Table 1; Fig. 5d–g). These included *Cxcl1* and *Cxcl10*, two chemokine ligands, as well as *Junb*, *Egr1,* and *Lin28a*. All of these transcripts have been reported as markers of quiescence^34–38^. Moreover, the key inhibitors of follicular emergence from a quiescent state *Smad 2* and *Smad3*^39,40^ were upregulated in NR5A2 cKO ovaries (Fig. 5f). To assess the downstream effect of this maintenance of quiescence, the cyclin-dependent kinase inhibitor, *Cdkn1a* was evaluated by qPCR. This transcript was substantially elevated in the cKO PND4 ovary (Fig. 5g). Together the evidence supports the view that NR5A2 influences the activation of primordial follicles, both by inducing a partial epithelial-to-mesenchymal transition and by regulating a decrease in quiescence regulators and an increase in regulators of cellular proliferation.

## Discussion

In the mammalian ovary, the progressive activation of the follicular reserve serves as the source of fertilizable ova during reproductive life. While progress has been made in recognition of the events in primordial follicle formation^41^, factors regulating their activation under physiological conditions remain less well understood. NR5A2, an orphan nuclear receptor, is well known to play essential roles in the ovary in: expansion of the cumulus oophorus^22^, ovulation^23^, granulosa cell proliferation^18^ and luteinization^24^. In the present study we show that NR5A2 is expressed in granulosa cells as early as the primordial follicle stage of follicle development. Based on this observation, we investigated the role of NR5A2 in early folliculogenesis in mouse ovaries between PND3 and PND6, i.e. during the window of time of termination of the formation of the primordial follicle pool^42^. Herein, we present evidence to indicate that there are two populations of primordial follicles in the mouse ovary, one quiescent where NR5A2 is absent, and a second, with NR5A2 expressed that is poised to activate and join the growing pool. Once follicles become activated, granulosa-specific expression of NR5A2 persists through follicular growth and ovulation.

In aid of confirming the follicle population in which NR5A2 is depleted, we demonstrated, by immunofluorescence and RNA in situ hybridization, that AMHR2 is expressed in all primordial follicles. This concurs with earlier studies showing AMHR2 in granulosa cells in the mouse ovarian reserve^43^. Indeed, it has been reported that the *Amhr2-*Cre allele is expressed even earlier in the mouse female gonad, i.e. by embryonic day E11.5–12.5^44,45^. The *Amhr2*-driven Cre recombinase has been employed in a prenatal knock-out mouse model to deplete NOTCH2 from germ cell nests^46,47^, and we had previously shown effective recombination and depletion in the ovary^23^. Therefore, we chose this model to explore the effects of NR5A2 on the early follicle population (*Nr5a2f.*^*/f*^; *Amhr2*^Cre+^). Herein we have demonstrated substantial and consistent (80–90%) depletion of *Nr5a2* in the PND4 mouse ovary.

In the cKO animals we observed a significantly smaller population of activated primary follicles at PND4, as indicated by the absence of expression of proliferation markers in the somatic cell component of the follicles. We hypothesized that the consequent increase in the number of primordial follicles observed in NR5A2 cKO mice ovaries results from a decreased rate of recruitment from a constant follicular pool. In support of this hypothesis, we showed that PND4 ovaries in organ culture displayed a larger population of primordial follicles and a reduced number of primary follicles.

PTEN is a well-established regulator of primordial follicle recruitment (reviewed in^48^). Not unexpectedly, this protein was substantially upregulated in the cKO follicle population at PND4. This finding is consistent with the role of PTEN and its downstream target FOXO3, the transcript of which was also upregulated in the cKO ovary, as the principal inhibitors of primordial follicle activation. Indeed, deletion of PTEN from oocytes of primordial follicles resulted in premature activation of the entire primordial follicle pool^49^. Similarly, studies have shown that *Foxo3* negative ovaries were enlarged by PND14 with greater numbers of early-growing follicles, and the primordial follicle reserve was totally depleted by that time^50,51^. Thus, one consequence of depletion of NR5A2 in primordial follicles is disruption of regulation of follicle activation normally exercised by the selective depletion of PTEN.

Molecular analysis has shown that in ovarian somatic cells, the transcription factor FOXL2 plays an important role in controlling both the formation and activation of primordial follicles. It dictates the differentiation of pre-granulosa into granulosa cells during primordial follicle recruitment^26,52^. In the present study, we show substantially reduced FOXL2 transcript and protein abundance in the NR5A2 cKO mouse, associated with the increased number of primordial follicles. This finding is consistent with the results of the germline depleting mutation of the *Foxl2* gene where the abrogation of the squamous to cuboidal transition of the primordial follicle granulosa cells following follicular activation was observed^26^. Further, our ChIPseq data in ovulatory adult mouse granulosa cells demonstrate peaks of NR5A2 binding associated with the *Foxl2* gene^53^. Although FOXL2 is expressed early in ovarian prenatal development^26^, the present findings indicate that it is likely downstream of NR5A2 in follicular activation in the postnatal primordial follicle.

To further identify NR5A2-regulated factors that may contribute to the transition from primordial to primary follicles, a comparison was made of our RNA sequencing results with a study of these two developmental strata from human ovarian tissue, in which granulosa cell-specific transcripts were identified and analyzed^54^. Fourteen transcripts that were greater in or unique to primordial follicles in that study^54^ were found to vary between the NR5A2 cKO and CON ovary in the present study. Among these, eleven were in greater abundance in cKO than CON (Supplementary Fig. S2). Several were regulators of ion transport or ion balance, including *Atp8a2*, *Gabre*, and *Slc30a3*, while several others were regulators of lipid binding or metabolism, including *Pla2g4c*, *Osbp2*, and *Atp8a2*. Wnt signaling is a positive regulator of both granulosa cell proliferation^55^ and epithelial-to-mesenchymal transition (reviewed in^56^). The suppressor of Wnt signaling, *Tle2*^57^ was more abundant in primordial follicles and in cKO ovaries. Functional annotations of all mRNA that were common to the present dataset and the study of Ernst et al.^54^ are in Supplementary Table S4.

A potentially counterintuitive finding in the RNA sequencing study was the observation that a number of meiosis-related, oocyte-specific transcripts were upregulated in cKO ovaries. It is unlikely that oocytes were meiotically active on PND4; thus, it is more likely that these differences reflect the abundance of oocytes rather than a difference in meiotic activity. Among oocyte-specific transcripts that were upregulated were well-established oocyte activation signals, including *Gdf9*, *Kit*, and *Figla*. This indicates that the ability of the oocyte to provide a signal for activation remains, or is perhaps increased in the absence of NR5A2, despite an impaired ability of granulosa cells to respond to the signal.

NR5A2 depletion has been shown to decrease granulosa cell proliferation by downregulating genes such as cyclins D and E, whose expression has been demonstrated to be depleted in quiescent cells^18,37^. Additional support for a reduced rate of activation of primordial follicles comes from evaluation of quiescence markers. For example, the abundance of the transcript for *Cdkn1a*, the cyclin dependent kinase inhibitor, is increased in PND4 cKO mice in the present study, consistent with its regulation by NR5A2 in breast cancer cells^58^. This protein is known to induce and maintain cell cycle arrest under conditions such a senescence or quiescence^59,60^. Further, it is a direct transcriptional target of NR5A2 in other tissues^18^. The chemokine ligands CXCL1, CXCL10 as well as EGR1, JUNB and LIN28A are associated with mitotic quiescence^34–37^. All five of these factors were significantly upregulated at the mRNA level in NR5A2 cKO ovaries at PND4. SMAD2 and 3 are key signal transducers in the TGF-beta signaling pathway. Their expression is associated with follicular quiescence while their loss was associated with activation^40^. SMAD3 directly maintains quiescence of granulosa cells by suppressing expression of the proto-oncogene MYC^39,61^. Both *Smad2* and *Smad3* were greater in cKO than CON ovaries, while *Myc* declined. Together, the gene expression patterns support the hypothesis that NR5A2 depletion from primordial follicles diminishes the rate of recruitment of primordial follicles into the growing follicle pool.

A key finding in the present study is that the presence or absence of NR5A2, defines two subsets of follicles in the primordial follicle population. We explored these two subpopulations, those in which NR5A2 was expressed in the pre-granulosa cells and those where it was not present. Neither expressed the mitotic marker KI-67, confirming their quiescence. The concept of more than one subset of primordial follicles is not new, as previous studies of the developmental chronology of follicle development identified two distinct populations of primordial follicles^47,62^ and lineage tracing has defined an initial small wave of follicles that populates the medulla of the ovaries^27^. These arise from bipotential cells in the stroma of the ovary, and begin to express Foxl2 in late embryonic life. The second population is of surface epithelial provenance and in these cells, robust Foxl2 expression only begins after birth. The follicle of the first wave exists in the ovaries for around three months and contribute to the onset of puberty. The second wave of primordial follicles, from the ovarian surface, replaces the first wave of follicles, providing fertility until the end of reproductive life. In the present study, the ovaries of CON mice collected at PND4 and cultured for ten days showed extensive activation of the medullary follicle population, and this activation was nearly absent in the cKO mice. Thus, the differential expression of NR5A2 in primordial follicles may be related in part to their location in the postnatal ovary, which appears to be a consequence of the respective provenance of the follicles^27^. Nonetheless, our in situ analyses also identified populations of primordial follicles in the cortex of more mature ovaries that either express or are devoid of NR5A2. Given the multiple roles of NR5A2, including induction of cell proliferation, we speculate that the follicles in which NR5A2 is absent are quiescent, while those expressing NR5A2 are in line for activation.

Finally, primordial follicle activation is characterized by a partial transition of granulosa cells from epithelial to mesenchymal state^63^. Studies show that NR5A2 promotes migration and invasion in breast cancer through up-regulating MMP9 and inactivating E-cadherin^30,58^. Together these findings suggest that NR5A2 is involved in epithelial mesenchymal transition^64^. Here we showed that the depletion of NR5A2 decreases pathways related to migration, increases the quantity of transcript of epithelial markers, *Cdh1* and *Gja1*, and decreases mesenchymal state related genes, *Snail1* and *Mmp9*. These findings provide another mechanism by which NR5A2 may regulate primordial follicle activation.

The downregulation of transcripts associated with migration in cKO ovaries was not limited to markers of epithelial to mesenchymal transition. As discussed previously, there is evidence for localization of growing follicles to the ovarian medulla, with primordial follicles remaining in the more rigid cortex. This process is not fully understood, but seems to be regulated in part by reorganization of the extracellular matrix^65^. In particular, actin polymerization, via the HIPPO pathway^66^, is a regulator of follicular activation. In the present study, *Coro1a* and *Synpo2*, both accessory to actin reorganization, were in reduced abundance in NR5A2-depleted ovaries.

The alterations in transcripts related to migration could also be indicative of changes in vascularization regulated by NR5A2 as regulators of vascular development, including *Mmp9*, *Fgfbp1*, and *Angptl7* were less in NR5A2 cKO ovaries. These results are consistent with some recent evidence of a role for vasculature in follicular activation^61^.

As noted above, the consequence of this NR5A2 depletion was a significantly greater number of primordial follicles in PND4 cKO mice, an increase that persists at least until PND13, when the follicle population is composed of primordial, primary and secondary but no antral follicles^28^. The disappearance of the increased population of primordial follicles in the cKO mice in adulthood may be related to the existence of census mechanism in the ovary by which an excess number of primordial follicles around birth is detected and removed from the ovary by adulthood. This concept is consistent with the resultant phenotype of BCL2 cKO mice where depletion was effected by the cKIT promoter^67^. These mice have more primordial follicles at birth but this increase is not maintained in postnatal life, and by postnatal day 30–60 there is no longer a difference^67^. Herein we show a substantial increase in the number of atretic follicles, frequently recognized by fragments of zonae pellucidae^50^. It is therefore possible that long-term blockade of primordial follicle growth in the cKO ovary results in clearance of the non-growing follicles and depletion of the primordial follicle pool by early adulthood, as has been shown by abrogating AKT activation pathway through conditional deletion of PDK1 in the oocytes^13^.

In our transgenic mouse model, NR5A2 is depleted by as much as 90%. Its residual expression and a potential compensation from the other member of the NR5A nuclear receptor family, NR5A1, also expressed in granulosa cells^68^ could explain the absence of phenotype in post-pubertal animals relative to the number of primordial follicles. As mature NR5A2 cKO mice present growing follicles and no sign of primary ovarian insufficiency, it may be that the excess number of primordial follicles is limited to a certain window of the developmental trajectory.

**Figure 6:**
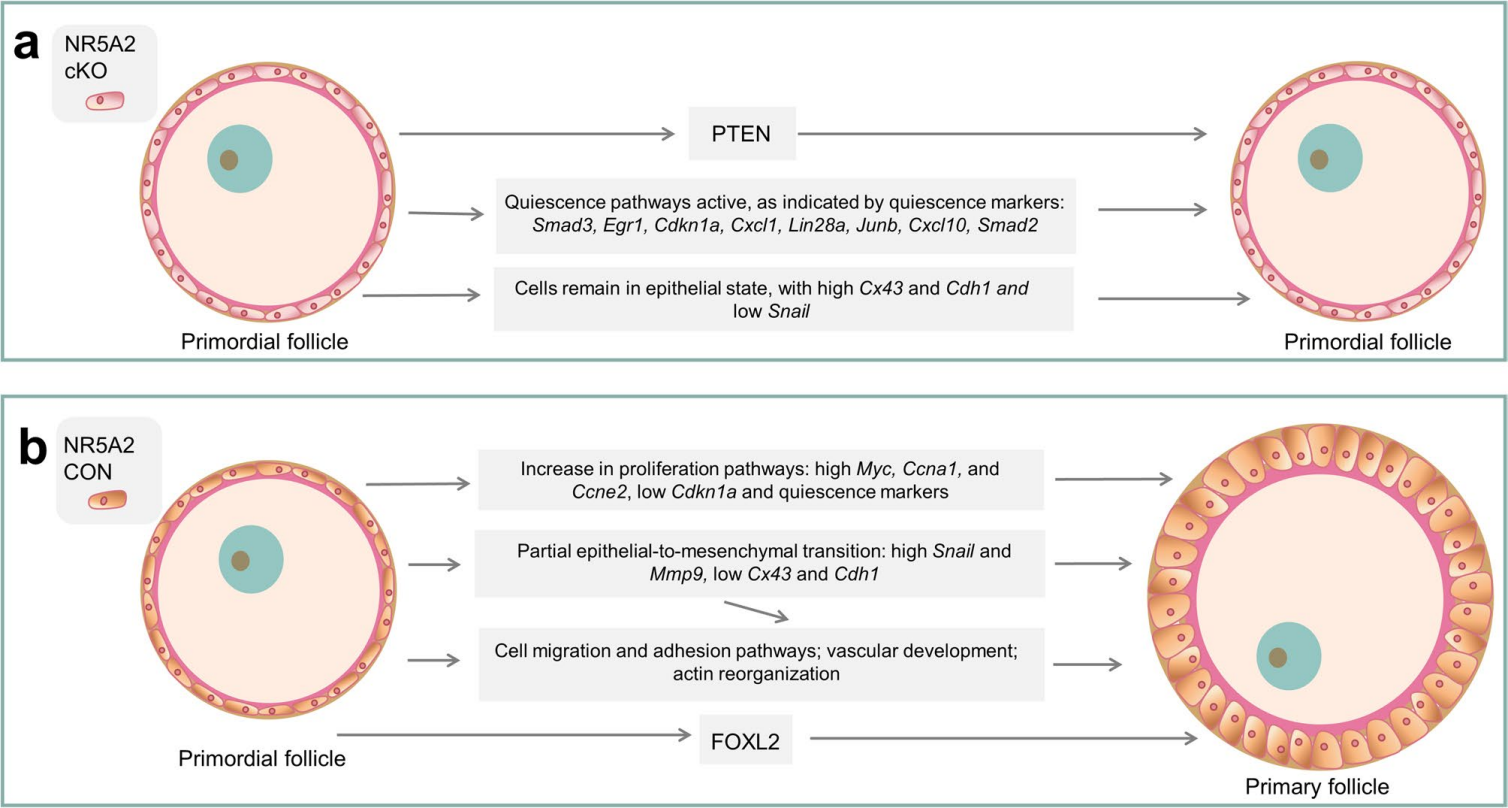
Mechanisms by which NR5A2 regulates follicular activation. (**a**) Mechanisms supporting the suppression of primordial follicle activation in follicles lacking NR5A2. (**b**) Mechanisms leading to activation of primordial follicles that express NR5A2.

In summary, we have demonstrated that depletion of NR5A2 from primordial follicles results in a larger ovarian reserve. We report a decreased rate of recruitment into the growing follicle pool, related to an increase of quiescence and a dysregulation of epithelial to mesenchymal transition (summarized in Fig. 6). We have further shown that NR5A2 is differentially expressed in populations of primordial follicles, and we conclude that this orphan nuclear receptor is an elemental generator of primordial follicle activation.

## Materials and methods

### Animals and colony maintenance

Animal experiments were approved by the University of Montreal Animal Care Committee and were conducted according to the guidelines of the Canadian Council on Animal Care. All mutant and control (CON) mice were maintained on the C57BL/6 background, under a 14-h light, 10-h dark cycle and provided food and water ad libitum. The *Nr5a2* floxed (*Nr5a2*^*fl/fl*^) mice have been described previously^21,23,69^. The LoxP sites in this line result in excision of exons 4 and 5 from the NR5A2 transcript^69^. Granulosa-specific depletion of NR5A2 was induced by crossing these animals with mice expressing Cre-recombinase driven by the anti-Mullerian type II receptor (*Amhr2*^*Cre/*+^)^21,23^ to produce conditional knockout (cKO) mice (genotype *Nr5a2*^*fl/fl*^; *Amhr2*^*Cre/*+^). Following DNA extraction from tails, littermates were genotyped. The genotyping primers bind to regions that are separated by one LoxP site in the region of intronic DNA between exons 3 and 4 of the *Nr5a2* gene. Therefore, the genotyping PCR resulted in a single, larger PCR product in mice homozygous for the LoxP site, a single, smaller PCR product in wildtype mice, and two products in mice heterozygous for the LoxP site. Control mice (CON) mice in these trials were nonmutant, *Nr5a2*^*fl/fl*^; *Amhr2*^*Cre/−*^ females^70^.

### Histology and follicle counting

Ovaries from postnatal day 4 (PND4), PND13 and mature (five-month old) animals were serially sectioned at 5 µM and stained with hematoxylin and eosin. For follicle counting, 3–9 ovaries were assessed per genotype and follicles were classified according to the following criteria: primordial follicles displayed a single layer of squamous pre-granulosa cells around the oocyte, primary follicles were defined as those with one layer of cuboidal granulosa cells, secondary follicles were those with more than one layer of cuboidal granulosa cells surrounding the oocyte, and antral follicles were the tertiary follicles presenting an antrum. Follicles with multiple layers of disorganized granulosa cells, non-homogeneous theca layer, damaged oocytes or fragments of zonae pellucidae^50^ were classified as atretic. For follicle enumeration in ovaries from PND4, PND13, and 5-month-old mice, multiple slides were counted for each ovary and data were expressed as an average number of unique follicles/slide. For cultured PND4 ovaries, only 1–2 slides/ovary were counted. Therefore, to normalize for differing section sizes, data were expressed as ratio of primordial to primary follicles and as percent of total follicles in the medulla. The Grubbs Outlier Test was performed and a single outlier was removed.

### RNA extraction and real-time PCR

RNA was extracted from postnatal day 4 (PND4) ovaries with PureLink RNA Mini-Extraction kit according to the manufacturer's instructions (Invitrogen, Waltham, MA). Reverse transcription was achieved using the SuperScript III reverse transcriptase (Invitrogen). Real-time quantitative polymerase chain reaction (qPCR) was performed using SsoAdvanced Universal SYBR Green Supermix (Bio-Rad Laboratories, Hercules, CA) with the CFX 96 Real-Time System, C1000 Touch Thermal Cycler (Bio-Rad Laboratories). The transcripts were amplified following the same cycling program: 30 s at 95 °C and then 40 cycles of 15 s at 95 °C and 30 s at 60 °C, followed by 5-s step of a 0.5 °C increase between 65 and 95 °C. Primers employed can be found in Supplementary Table S5. For *Nr5a2*, primers were designed within the floxed portion of the sequence for the *Nr5a2* transcript, with the forward primer in exon 3 and the reverse primer in exon 5.

### RNA in situ hybridization (RNAScope)

Tissue processing and embedding were performed using standard techniques. Tissues were fixed in 10% formalin overnight, paraffin embedded and cut into 5 μm sections. RNA in situ hybridization was performed with RNAscope 2.5 HD Detection Kit (RED) (Advanced Cell Diagnostics, Newark, CA) following manufacturer’s instructions and, as previously described^71^. The tissue sections were hybridized with probes spanning mouse *Amhr2* and human and mouse *Nr5a2* mRNA (Advanced Cell Diagnostics) in the HybEZ hybridization oven (Advanced Cell Diagnostics) for 2 h at 40 °C, following a series of pretreatment steps. The mouse Amhr2 probe (Advanced Cell Diagnostics catalog number 489821) targets nucleotides 914–1809. The human *Nr5a2* probe (catalog number 490261) targets nucleotides 773–1786 of the human sequence. The slides were then processed for standard signal amplification steps per manufacturer’s instructions. Red chromogen development was performed following the RNAscope 2.5 HD detection protocol. The slides were then counterstained in 50% hematoxylin for 2 min, air-dried and cover slipped with EcoMount (Biocare Medical, Pacheco, CA).

### Transcriptome sequencing and analysis

Ovaries from postnatal day 4 (PND4; n = 4 per time point) were collected and RNA purified as above. Quality of RNA was assessed using an Agilent Bioanalyzer (Agilent Technologies, Santa Clara, CA) and only samples of high quality were used. The library preparation was performed with a New England Biolabs mRNA stranded library preparation kit (Rowley, MA) and library QC followed. Pair-ended sequencing was performed at Génome Québec (Montreal, QC), according to their standard procedures with an Illumina HiSeq4000, generating 20,000,000 reads per sample of 100 base pairs per read. Reads with a quality score less than 20 were removed and alignment was performed using the STAR open source alignment tool^72^. The reference genome employed was the Genome Reference Consortium *Mus musculus* GRCm38 (mm10). To evaluate RNA abundance at the gene level, reads mapped to each gene were quantified using featureCounts. Normalization and analysis of differential abundance were performed using the package DEseq2 in R, with a Benjamini Hochberg False Discovery Rate (FDR) correction (FDR = 5%). Individual mRNA were considered significantly differentially abundant at Padj < 0.05 and fold change of at least 1.5 up or down (equivalent to log2 fold change of |0.585|). Pathway analysis was performed using Metascape^73^. For integration of these data with the dataset of Ernst et al., (2018), the data of Ernst et al., were accessed directly as reported and Microsoft Excel was used to find commonalities between the datasets. DAVID^74,75^ was used for functional annotation. Heatmaps were drawn in the program Morpheus, which is freely available online at https://software.broadinstitute.org/morpheus/.

### Fluorescent immunocytochemistry

Slides of formalin or paraformaldehyde-fixed paraffin-embedded PND4 ovaries from control and cKO mice were rehydrated as previously described^21^ and blocked 1 h with 5% bovine serum albumin in phosphate buffered saline with 0.1% Tween-20 (PBST). Slides treated for NR5A2 immunofluorescence were blocked for a further hour with mouse-on-mouse (MOM) blocking reagent (Vector Labs, Burlingame, CA). Slides were then incubated overnight at 4 °C with antibodies against FOXL2 (1:8000 in BSA5%/PBST 0.1%; a generous gift from Dr. D. Bernard), NR5A2 (1:200 in MOM kit dilution reagent, Vector Labs), AMHR2 (1:200 in BSA5%/PBST 0.1%, R&D Systems, Minneapolis, MN), PTEN (1:200 in BSA 5%/PBST 0.1%, Cell Signaling Technology), DDX4 (1:200 in BSA 5%/PBST 0.1%, Invitrogen) or KI-67 (1:200 in BSA5%/PBST 0.1%, Abcam, Cambridge, UK). For double staining with NR5A2 and KI-67, both antibodies where diluted in M.O.M. kit dilution reagent. CY3 conjugated anti-mouse (Jackson ImmunoResearch Laboratories, West Grove, PA) diluted 1:400 in BSA 5%/PBST 0.1% was used for NR5A2 and AMHR2 immunofluorescence. CY3 conjugated goat anti-rabbit IgG (Jackson ImmunoResearch Laboratories) was used as second antibody for PTEN, FOXL2, cleaved caspase 3 and KI-67 immunolocalization, at the same concentration. Finally, slides were counterstained with DAPI, 1:1000 in PBS for 5 min before being washed three times in PBS and mounted with Permafluor. CellProfiler version 4.06 Software^76^, which is freely available at www.cellprofiler.org, was used to quantify signal intensity, by the following method: first, the color images were converted to grayscale by splitting, to conserve only the main channel (blue for DAPI, red for NR5A2 and PTEN immunofluorescence, and green for FOXL2). Based on DAPI images, the follicles or areas of interest were then manually delimitated and the intensity of the staining quantified. Finally, the controls were normalized to 1 and cKO intensity was calculated relative to 1.

### Culture of neonatal ovaries

Postnatal day 4 (PND4) mice ovaries from CON and cKO animals were dissected and placed in 50 μl drops of medium and cultured for 10 days on 0.4 μm floating filters at 37 °C in a chamber containing 5% CO_2_. Filters were placed in four-well culture plate on 0.5 ml DMEM-F12 medium supplemented with 0.1% Albumax, penicillin–streptomycin, 0.1% fetal bovine serum, 27.5 μg/ml transferrin, and 0.05 mg/ml L-ascorbic acid^77,78^. Culture medium was changed every second day. After 10 days of culture, ovaries were fixed for 4 h in 4% PAF, washed with PBS and mounted in blocks before being immunostained with DDX4 antibody to facilitate follicle counting.

### Statistical analyses

All values are expressed as mean ± SEM. For all experiments other than the transcriptomics experiment, statistical comparisons were made using Student’s t-test with Welch’s correction on GraphPad Prism V7.0 (Graphpad Software Inc., San Diego, CA). Percentage data were arcsine transformed prior to analysis.

## Supplementary information

Supplementary Figures.

Supplementary Table 1.

Supplementary Table 2.

Supplementary Table 3.

Supplementary Table 4.

Supplementary Table 5.
