# Supplementary material for: A role for orphan nuclear receptor liver receptor homolog-1 (LRH-1, NR5A2) in primordial follicle activation
Source: Sci Rep. 2021 Jan 13;11:1079. doi: 10.1038/s41598-020-80178-4 (PMC7807074; doi:10.1038/s41598-020-80178-4)
Supplement: Supplementary file 1 — Supplementary Figures. [file 41598_2020_80178_MOESM1_ESM.pdf]

A Role for Orphan Nuclear Receptor Liver Receptor Homolog-1 (LRH-1, NR5A2) in Primordial Follicle Activation

Marie-Charlotte Meinsohn, Camilla H. K. Hughes, Anthony Estienne, Hatice D. Saatcioglu, David Pépin, Raj Duggavathi, and Bruce D. Murphy

Supplementary Figures

Supplementary figure S1

a

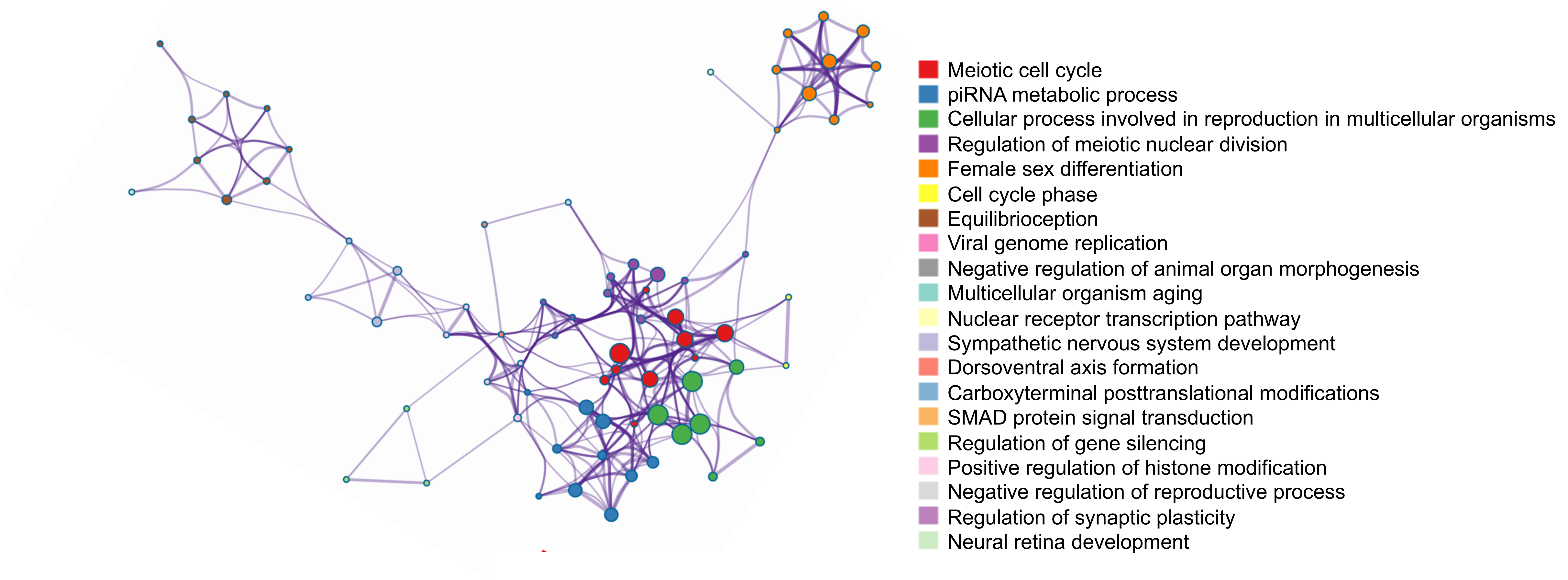

b

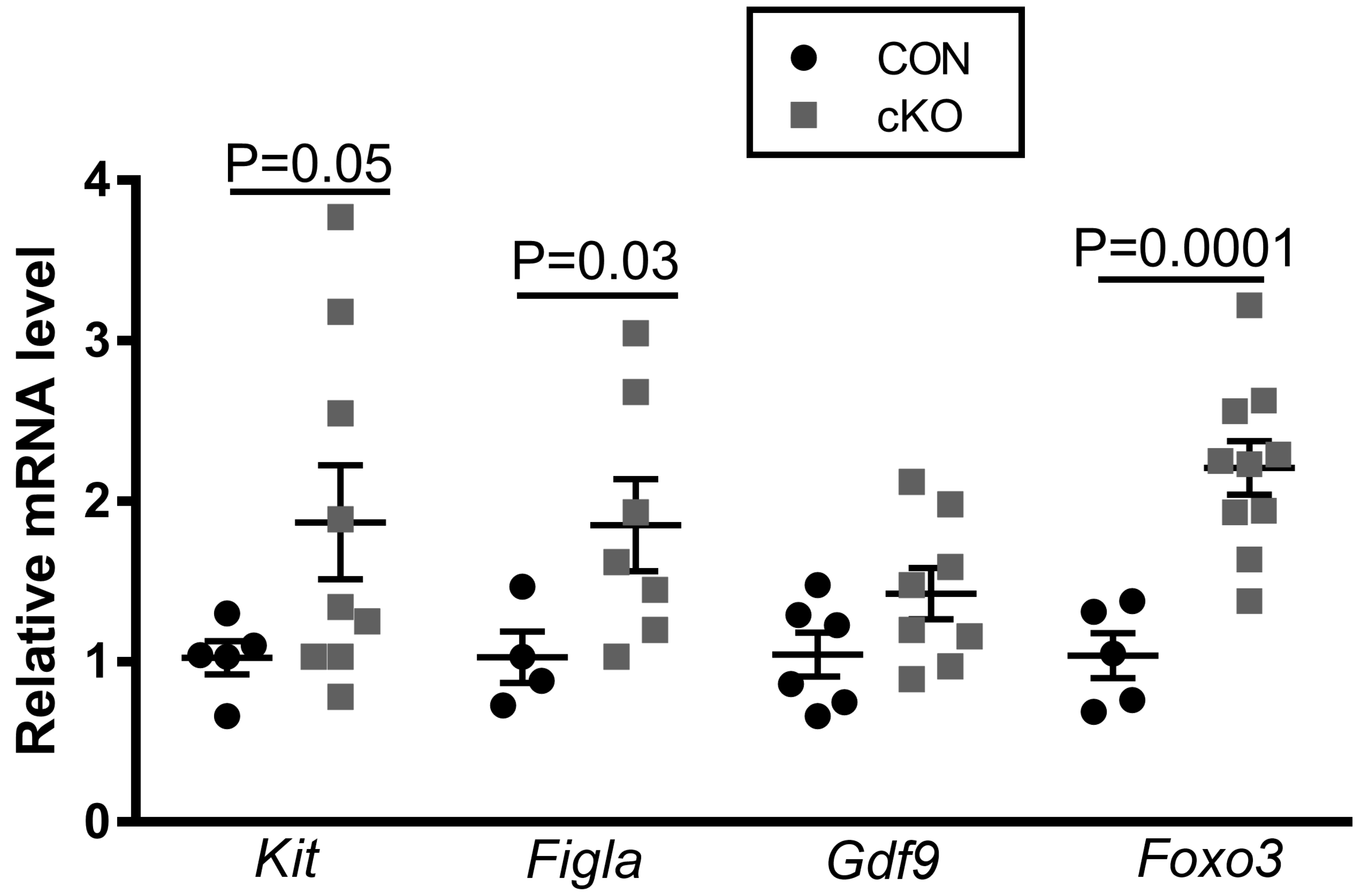

Supplementary figure S1. Transcripts that were upregulated by NR5A2 cKO. a) Pathway analysis transcripts that were upregulated by by NR5A2 cKO. The color of each circle represents the functional cluster, while the edges represent the similarity or relatedness among clusters. b) Abundance of oocyte-specific transcripts that changed (Padj < 0.05; Kit, Gdf9, Foxo3) or tended to change (P < 0.05; Figla) by RNAseq, as measured by qPCR in CON and cKO murine ovaries at PND4 (n=5-10 animals per genotype).

Supplementary figure S2

a

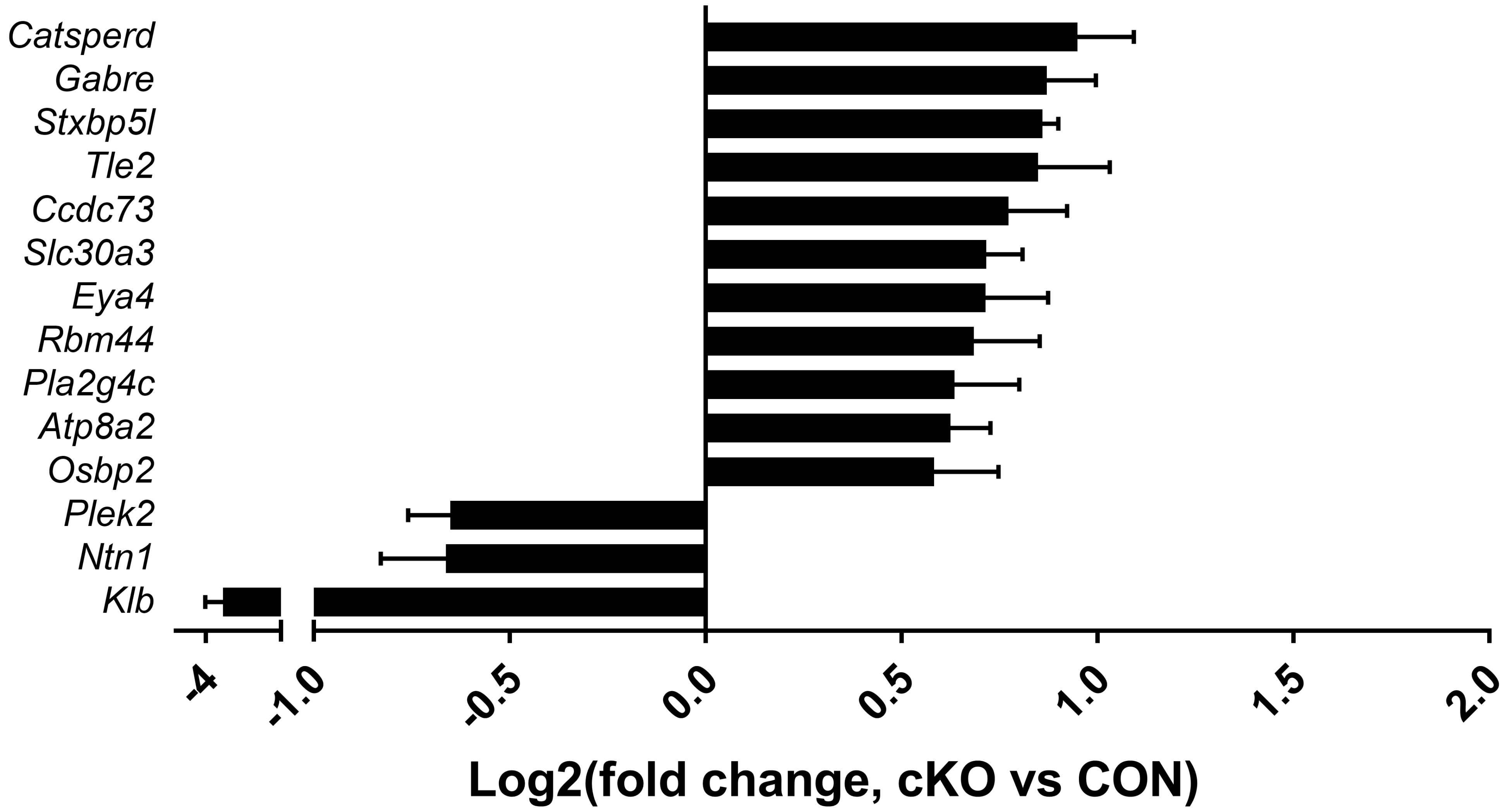

b

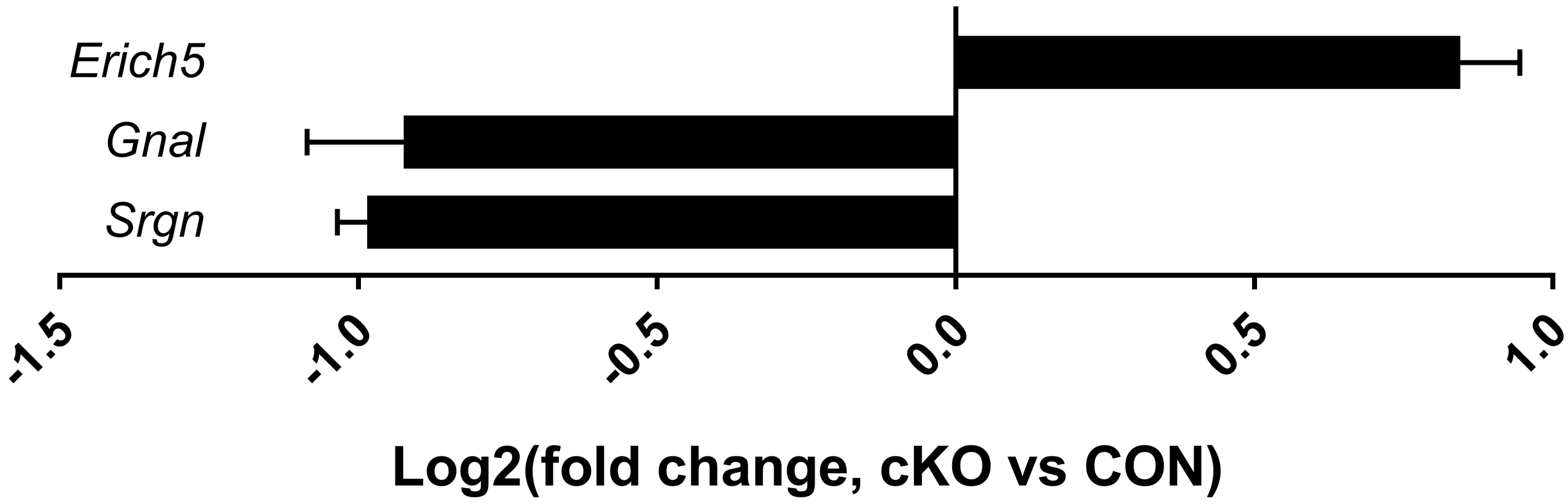

Supplementary figure S2. mRNA altered by NR5A2 conditional knockout and during the primordial-to-primary follicle transition. (a) mRNA that were more abundant in primordial follicles (Ernst et al., 2018) and were regulated by NR5A2 cKO. (b) mRNA that were more abundant in primary follicles (Ernst et al., 2018) and were regulated by NR5A2 cKO.
